# Oncology nutrition care in Indonesia: a cross-sectional survey to characterise practices, barriers and enablers among nutritionists and dietitians

**DOI:** 10.3389/fnut.2026.1756083

**Published:** 2026-04-30

**Authors:** Choirun Nissa, Lauren Hanna, Judy Bauer

**Affiliations:** 1 Department of Nutrition, Dietetics and Food, Monash University, Notting Hill, VIC, Australia; 2 Department of Nutrition Science, Faculty of Medicine, Diponegoro University, Semarang, Central Java, Indonesia

**Keywords:** barrier, cancer, dietitian, enabler, Indonesia, nutritionist, nutrition care

## Abstract

**Purpose:**

Nutrition care is essential for improving oncology treatment outcomes, however evidence on current practices and adherence to the Nutrition Care Process framework in Indonesia is scarce. This study explored oncology nutrition care practices, identified barriers, enablers and behavioral determinants influencing evidence-based practice experienced by Indonesian nutritionists and dietitians.

**Methods:**

Based on the Theoretical Domain Framework (TDF) and Capability Opportunity Motivation – Behavior (COM-B) system, a 32-item survey was developed to characterise nutrition care practice, identify barriers and enablers, and the behavioral determinants influencing evidence-based oncology nutrition care in Indonesia. The cross-sectional survey was distributed online to members of the Indonesian Dietitian Association.

**Results:**

268 participants (91% female; 67.5% nutritionists, median age 34 (interquartile range 28–41) years) met eligibility criteria and completed the survey. Participants reported high confidence, being motivated by a strong professional identity and personal satisfaction. Awareness of international evidence-based oncology nutrition guidelines was low (ranging from 11.2 to 44.8%), most of which are only available in English. Individual components of nutrition assessment were completed by most participants (anthropometry 91.8%, biochemical data 88.4%, physical examination 71.3%, and dietary intake 95.1%); however, only 55.6% used a validated malnutrition assessment tool. Key enablers to providing nutrition care included access to general nutrition training (64.2%) and peer support (79.9%), while major barriers were lack of oncology-specific nutrition training (75.7%) and limited access to guidelines in the Indonesian language (42.9%).

**Conclusion:**

Despite strong motivation, gaps in capability and opportunity hinder evidence-based oncology nutrition care in Indonesia. Implementation strategies to address these gaps include development of culturally adapted Indonesian oncology nutrition care guidelines, integration of validated malnutrition assessment tools into practice, and implementation of accessible cost-effective oncology-specific nutrition training. These strategies are essential to strengthen evidence-based practice and improve patient outcomes in Indonesia.

## 1 Introduction

Cancer is one of the most prevalent non-communicable diseases in Indonesia, with over one million people diagnosed over a five-year period according to the Global Cancer Observatory (Globocan) 2022 report (1). During cancer treatment (surgery, radiation and/or chemotherapy), patients are at high risk of malnutrition due to symptoms and treatment side effects which limit food intake (e.g., anorexia, taste alteration, nausea, vomiting, dysphagia, pain), compounded by cancer-related metabolic changes (2). In Indonesia, data from clinical settings indicate that malnutrition affects 33–78% of patients with cancer (3, 4), with this wide prevalence range reflecting differences in cancer diagnoses, treatment and nutrition assessment methods used. Malnutrition is associated with increased postoperative complications and hospital length of stay, reduced treatment completion, poorer quality of life and higher mortality (2, 3, 5–7). Individualized nutritional support provided by nutritionists and dietitians can improve both patient and clinical outcomes (8–10).

The Nutrition Care Process (NCP) framework was developed by the Academy of Nutrition and Dietetics (2003) to reduce variation in nutrition care and promote a predictable outcome regardless of location and resources (11). It consists of four interrelated steps: nutrition assessment, diagnosis, intervention, and monitoring and evaluation (11). In 2006, the NCP was introduced into Indonesian dietetic practice by the Indonesian Dietitian Association (12, 13) and was established as a national standard under Ministry of Health’s regulations in 2013 (13). However, the uptake and implementation of the NCP in the oncology setting in Indonesia remain poorly described, and to date, there are no published data detailing routine oncology nutrition care practices since its introduction. Our recent scoping review (2024) characterised oncology nutrition care practices in Southeast Asia; however, no studies specifically described current practice in Indonesia (14).

Implementing the NCP into routine clinical practice is challenging, and influenced by multiple factors such as staffing levels, the hospital’s nutrition care system, access to education and training, availability of guidelines, past work experience, involvement of professional associations, managerial support, and workplace culture (14–16). Identifying the factors influencing NCP implementation is an essential step to improve patient outcomes in the long term. Given the central role of human behavior in implementation, behavior informed approaches can enhance the adoption of evidence-based practice (17). Implementation science frameworks provide structured methods to understand and enhance the adoption of evidence-based practices in real-world settings. The Theoretical Domains Framework (TDF), developed through a synthesis of multiple behavior change theories, presents a systematic method for identifying a wide range of behavioral determinants over 14 domains (18). Complementing this, the Capability Opportunity Motivation - Behavior (COM-B) system links these determinants to core components of behavior: capability, opportunity and motivation (19). Together, these frameworks can inform the design and evaluation of implementation strategies tailored to a specific practice setting.

Research utilizing these behavioral frameworks to explore dietetic practice is limited. Previous studies have applied the TDF to identify barriers and enablers in specific aspects of the NCP in non-cancer populations (17, 20, 21) and in cancer screening or public health programs (22–24). However, no studies have applied an implementation science framework to explore the barriers and enablers to implementing nutrition care for patients with cancer. Therefore, the primary objectives of this study were to explore current clinical nutrition care practices and to identify the barriers and enablers experienced by Indonesian nutritionists and dietitians in managing patients with cancer using the TDF and COM-B frameworks to inform the design of future implementation strategies. The secondary objectives were to compare nutrition care practice and barriers or enablers by profession (nutritionist or dietitian) and geographical location.

## 2 Materials and methods

### 2.1 Study design and ethics

This cross-sectional study was conducted from July to August 2024. The study protocol was reviewed and approved by the Monash University Human Research Ethics Committee (Project ID 42397, June 2024). Prior to providing consent, participants received an explanatory statement outlining the aims, consent, withdrawal, possible benefits and risks, and contact information for the researchers. Informed consent was obtained from all participants. This research is reported according to the Strengthening the Reporting of Observational Studies in Epidemiology (STROBE) guideline (25).

### 2.2 The survey

**Figure 1:**
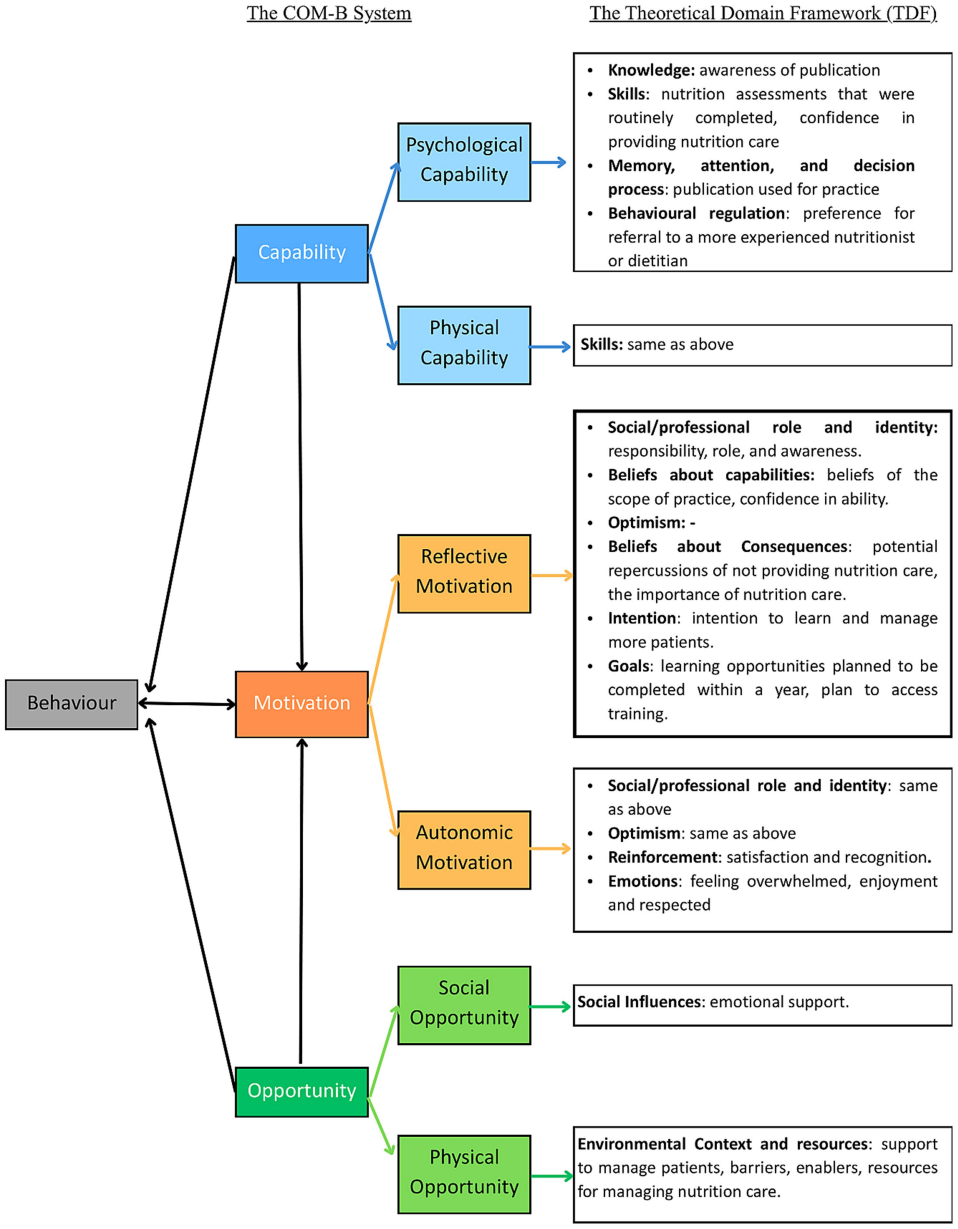
The capability opportunity motivation – behavior system and theoretical domain framework used for questionnaire development (19), adapted from (27), licensed under CC BY 4.0.

The 32-item survey was designed using the TDF to explore recent practices, barriers, enablers, confidence, beliefs and opinions, and to identify the influence of the TDF domains on behavior. The survey consisted of two sections: (1) socio-demographic information and professional characteristics, and (2) current and past practices, and perceived barriers and enablers related to the provision of nutrition care for patients with cancer. It was adapted from the original and validated TDF framework (18) using the template available for use in implementation research (26) tailored to the specific behavior and clinical context of oncology nutrition care, particularly the NCP (11). The survey included questions covering the four interrelated steps of the NCP (nutrition assessment, diagnosis, intervention, monitoring and evaluation), as well as related activities such as nutritional screening and post-discharge nutritional care practices. Thirteen domains of the TDF were mapped to the COM-B system (19) to identify behavioral determinants influencing evidence-based oncology nutrition practice among nutritionists and dietitians; ‘optimism’ was the only domain not used in the questionnaires. Agreement with each statement was measured using a 10-point Likert scale, ranging from one (strongly disagree) to ten (strongly agree). Figure 1 presents the COM-B system mapped to the TDF (18, 27) and corresponding questionnaire items.

### 2.3 Translation quality control and validation

At the request of the Indonesian Nutritionist Association, the primary body of the Indonesian Dietitian Association, the explanatory statement and survey were translated into Indonesian to ensure accessibility. The Translation, Review, Adjudication, Pre-test, and Documentation (TRAPD) method (28) was used to achieve cross-cultural content validity and linguistic equivalence. An independent bilingual dietitian first translated the English versions of the explanatory statement and questionnaire into Indonesian. Subsequently, three independent bilingual professionals with expertise in dietetics, nutrition or health education conducted back-translations into English to verify alignment with the original content and ensure linguistic accuracy, contextual and cultural appropriateness. All translations were reviewed by the research team, with revisions made at this stage. Items that raised concerns or lacked consensus proceeded to adjudication, where final decisions on wording, structure and readiness for pre-testing were made. The questionnaire was pre-tested in three rounds (first in English and then twice in Indonesian) to assess clarity, comprehension and acceptability for respondents. Face and content validity were evaluated during pre-testing with two English-speaking dietitians and twelve Indonesian-speaking nutritionists or dietitians. Feedback-informed revisions to wording, order and response options. The full process was documented for transparency and replicability.

### 2.4 Participant recruitment

The online survey was administered using Qualtrics (29) and distributed to members of the Indonesian Dietitian Association, targeting nutritionists and dietitians involved in the care of adult patients with cancer. Eligible participants were those currently or previously working with adult patients (≥ 18 years) diagnosed with cancer, had ≥ one-month’s experience in providing nutrition care, were fluent in Indonesian language and had internet access. Convenience sampling was used. At the time of recruitment, the Association had 4,145 members (30). Initial recruitment occurred during the Indonesian National Dietitians Workshop (Lombok, 18 July 2024) via flyers distributed to all workshop registrants and a short presentation by an author (CN), followed by broadcast messages (video and survey links) shared in the event WhatsApp group. To broaden reach, the flyer and video were also shared on social media. The survey included the explanatory statements, e-consent, eligibility screening, and main survey items, and remained open between 18 July to 16 August 2024. Only eligible respondents were granted access to the full survey.

### 2.5 Statistical analysis

Survey responses were exported from Qualtrics (29) and imported into the Statistical Package for Social Science (SPSS) version 29.0 (IBM Corp., Armonk, NY, USA) (31) for analysis. Normality of quantitative data was assessed using the Kolmogorov–Smirnov test. Between-group comparisons (profession: nutritionists, dietitians; geographical location: metropolitan/large urban, regional/small city, rural/remote) used non-parametric tests (Mann–Whitney, Kruskal-Wallis, Chi-Square) as appropriate. Bonferroni corrections were applied to control for Type 1 error. Significant Chi-Square results were followed by post-hoc pairwise comparisons to determine between-group differences. Statistical significance was set at *p* < 0.05. Listwise deletion was used for missing data. Open-ended responses on the role of the Indonesian Dietitian Association were synthesized using a prompt-generated word cloud created via Microsoft Copilot within the Monash University network.

## 3 Results

### 3.1 Demographic characteristic

**Figure 2:**
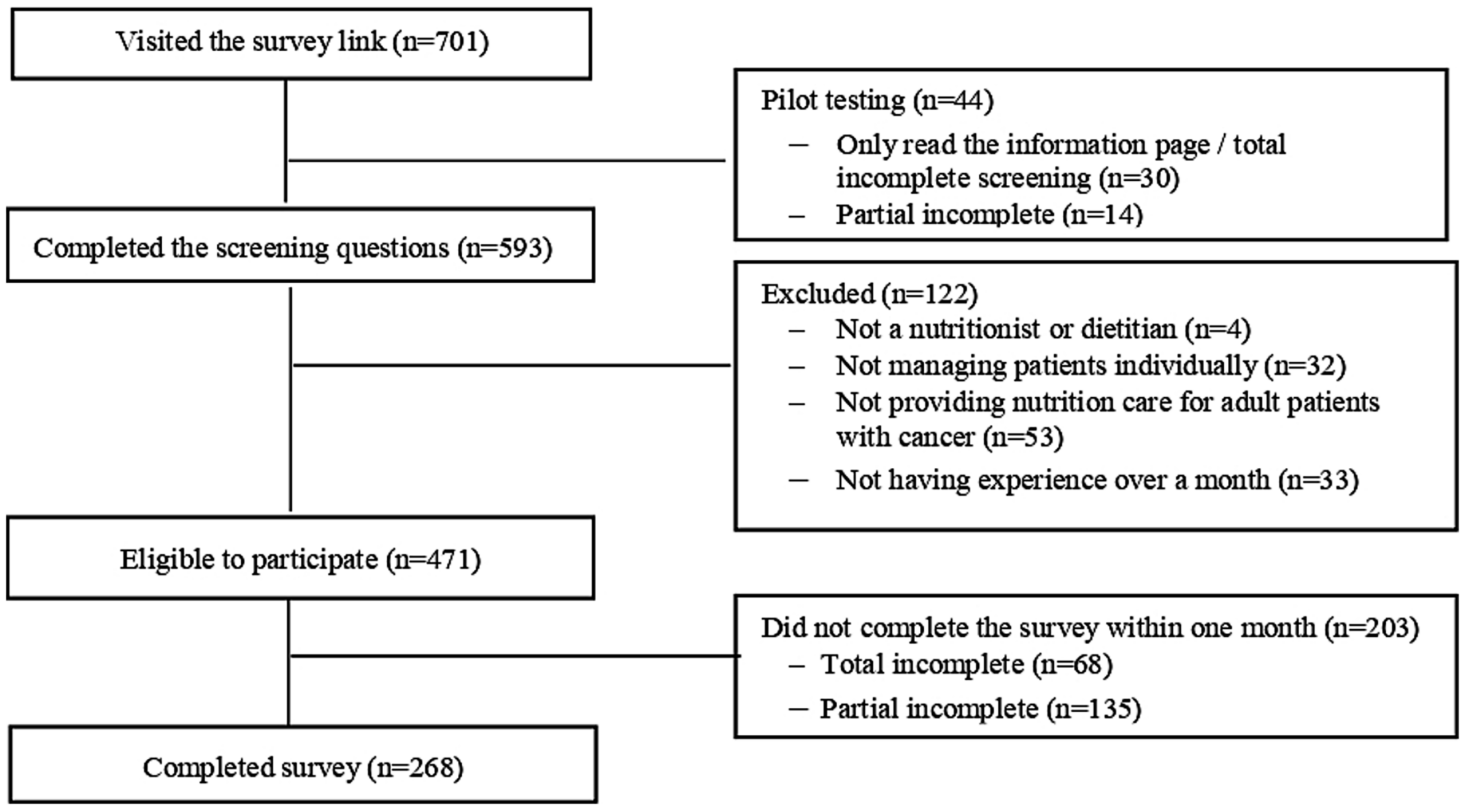
Flow diagram of study recruitment.

Of 593 individuals who completed screening, 268 met eligibility and fully completed the survey (Figure 2). Most participants were female (91.4%), with a median age of 34 (IQR 28–41) years. Approximately three-quarters (73.9%) were based in the Java-Bali islands, working primarily in metropolitan or large urban areas (52.2%) or regional/small cities (45.1%). The majority held a bachelor’s degree (62.3%), identified as nutritionists (67.5%) and were employed full-time in government hospitals (71.6%), and predominantly worked in inpatient care (91%). Oncology was reported as the fifth most common area of regular clinical practice (38.4%). Over half of the participants had less than six years of professional experience (65.7%) and reported providing nutrition care to patients with cancer at least monthly (63.4%) (Table 1).

**Table 1:** Socio-demographic and characteristics of participants (*n* = 268).

Characteristics	*n* (%)
Profession	
Nutritionist	181 (67.5)
Dietitian	86 (32.1)
Area of work	
Java and Bali	198 (73.9)
Sumatra	34 (12.7)
Borneo	15 (5.6)
Sulawesi	11 (4.1)
Nusa Tenggara, Maluku, Papua	10 (3.7)
Geographical location	
Metropolitan or large urban area	140 (52.2)
Regional or small city	121 (45.1)
Rural or remote	6 (2.2)
Highest nutrition educational background	
Bachelor’s /Applied bachelor’s degree	167 (62.3)
Diploma (I, II, III)	45 (16.8)
Dietetics professional education	27 (10.1)
Master’s degree	27 (10.1)
Doctoral degree	1 (0.4)
Primary work setting	
Government hospital	192 (71.6)
Private hospital	60 (22.4)
Primary/community health (Public health centre and primary clinic)	6 (2.2)
Cancer care facility/cancer hospital	5 (1.9)
Clinic	3 (1.1)
Other	1 (0.4)
Primary practice area	
Inpatient dietetics care	244 (91.0)
Outpatient dietetics care	9 (3.4)
Community/public health nutrition	4 (1.5)
Other	10 (3.7)
Clinical areas	
Endocrinology	179 (66.8)
Surgery	130 (48.5)
Nephrology	116 (43.3)
Cardiology	113 (42.2)
Oncology	103 (38.4)
Neurology	102 (38.1)
Geriatrics	95 (35.4)
Infectious disease	82 (30.6)
Critical care	78 (29.1)
Respiratory	77 (28.7)
Gastroenterology	70 (26.1)
Eating disorders and mental health	40 (14.9)
Allergy and immunology	34 (12.7)
Disability	17 (6.3)
Other	59 (22)
Employment hours	
Full-time (40 h/week or more)	208 (77.6)
Part-time (30–39.9 h/week)	46 (17.2)
Part-time (20–29.9 h/week)	6 (2.2)
Part-time (<20 h/week)	5 (1.8)
Not currently working	2 (0.7)
Experience in working with adult patients with cancer	
≤1 year	53 (19.8)
2–5 years	123 (45.9)
6–10 years	53 (19.8)
11–19 years	25 (9.3)
20 years or more	12 (4.5)
Frequency in working with adult patients with cancer	
Daily	53 (19.8)
Weekly	41 (15.3)
Monthly	76 (28.4)
Quarterly	38 (14.2)
Rarely, i.e., once or twice a year	58 (21.6)
Number of adult patients with cancer managed in the last month	
1–50 patients	133 (49.6)
51–100 patients	12 (4.5)
>100 patients	10 (3.7)
No response	23 (8.6)
Not relevant (not currently working with patients with cancer)	90 (33.6)

### 3.2 COM-B: capability, opportunity, motivation

A summary of survey responses, along with comparisons by profession and geographical location mapped to the COM-B system are presented in Table 2 (Capability domain), Table 3 (Opportunity domain) and Table 4 (Motivation domain).

**Table 2:** Practices and perceived barriers or enablers to the provision of nutrition care for patients with cancer by Indonesian nutritionists and dietitians: survey questions addressing domains of the TDF mapped to the capability aspect of the COM-B system.

Theoretical domain framework	All (*n* = 268)	Nutritionists/dietitians			Geographical location			
		Nutritionist (*n* = 181)	Dietitian (*n* = 86)	*p*-value	Metropolitan/large urban area (*n* = 140)	Regional or small city (*n* = 121)	Rural or remote (*n* = 6)	*p*-value
Knowledge								
**Awareness of publication, *n* (%)**								
Hospital Nutrition Service Guidelines (Regulation of The Minister of Health of The Republic of Indonesia Number 78 of 2013)	251 (93.7)	167 (92.3)	83 (96.5)	0.184	126 (90.0)	119 (98.3)	6 (100)	**0.015** ^a*^
International Dietetics and Nutritional Terminology (IDNT) Reference Manual: Standard Language for the Nutrition Care Process 4th Edition (2013)	165 (61.6)	113 (62.4)	51 (59.3)	0.624	91 (65.0)	70 (57.9)	4 (66.7)	0.481
eNCPT (2020)	160 (59.7)	98 (54.1)	62 (72.1)	**0.005**	85 (60.7)	71 (58.7)	4 (66.7)	0.891
ESPEN guidelines on nutrition in cancer patients	120 (44.8)	74 (40.9)	45 (52.3)	0.079	70 (50.0)	49 (40.5)	1 (16.7)	0.102
ESPEN practical guideline: Clinical Nutrition in cancer	118 (44.0)	71 (39.2)	46 (53.5)	**0.028**	67 (47.9)	48 (39.7)	2 (33.3)	0.359
Technical guidelines on adult Palliative patients with cancer (Ministry of Health)	86 (32.1)	51 (28.2)	35 (40.7)	**0.041**	54 (38.6)	31 (25.6)	1 (16.7)	0.056
The American Society for Parenteral and Enteral Nutrition (ASPEN) guidelines	74 (27.6)	40 (22.1)	33 (38.4)	**0.005**	45 (32.1)	28 (23.1)	1 (16.7)	0.218
Oncology Evidence-Based Nutrition Practice Guideline for Adults (Academy of Nutrition and Dietetics)	42 (15.7)	19 (10.5)	22 (25.6)	**0.001**	20 (14.3)	20 (16.5)	2 (33.3)	0.431
Position statement of cancer-related malnutrition and sarcopenia (Clinical Oncology Society of Australia)	30 (11.2)	10 (5.5)	19 (22.1)	**<0.001**	18 (12.9)	10 (8.3)	2 (33.3)	0.112
I am not aware of these publications	4 (1.5)	4 (2.2)	0 (0)	0.309	4 (2.9)	0 (0)	0 (0)	0.074
Other	13 (4.9)	8 (4.4)	5 (5.8)	0.762	7 (5.0)	6 (5.0)	0 (0)	0.855
Skills								
**Nutritional assessments that were routinely completed for patients with cancer, *n* (%)**								
Anthropometry assessment	246 (91.8)	169 (93.4)	77 (89.5)	0.277	128 (91.4)	113 (93.4)	6 (100)	0.652
Biochemical data review	237 (88.4)	158 (87.3)	78 (90.7)	0.417	123 (87.9)	111 (91.7)	3 (50.0)	**0.006** ^b**, c***^
Nutrition-focused physical examination, including muscle and fat stores, fluid status, overall appearance	191 (71.3)	125 (69.1)	65 (75.6)	0.272	106 (75.7)	81 (66.9)	4 (66.7)	0.283
Dietary intake assessment	255 (95.1)	171 (94.5)	83 (96.5)	0.558	132 (94.3)	118 (97.5)	5 (83.3)	0.157
Malnutrition assessment using a validated tool	149 (55.6)	96 (53.0)	53 (61.6)	0.187	81 (57.9)	65 (53.7)	3 (50.0)	0.766
The presence of nutrition impact symptoms related to cancer	203 (75.7)	135 (74.6)	68 (79.1)	0.423	111 (79.3)	88 (72.7)	4 (66.7)	0.404
Effect of specific treatments and prescribed medications	134 (50)	85 (47.0)	49 (57.0)	0.126	79 (56.4)	52 (43.0)	2 (33.3)	0.067
Nutritional intake through tube feeding (if relevant)	214 (79.9)	140 (77.3)	73 (84.9)	0.152	115 (82.1)	95 (78.5)	4 (66.7)	0.560
Quality of life	147 (54.9)	94 (51.9)	53 (61.6)	0.137	80 (57.1)	64 (52.9)	3 (50.0)	0.764
Other	20 (7.5)	8 (4.4)	12 (14.0)	0.006	15 (10.7)	5 (4.1)	0 (0)	0.103
Confidence in providing nutrition care								
Conduct a nutritional assessment of a patient with cancer and identify main signs/symptoms related to malnutrition	8 (7–9)	8(7–9)	9 (8–10)	**0.006**	9 (8–10)	8 (7–9)	7.5 (7–8.5)	**0.003** ^d*^
Provide nutrition diagnosis and identify problem-etiology-sign/symptoms within individual patients	8 (7–9)	8(7–9)	8 (8–10)	0.067	9 (8–10)	8 (7–9)	8 (7–8.5)	**0.013** ^e*^
Provide several nutritional recommendations to address the identified nutritional problem	8 (8–9)	8 (8–9)	8 (9–10)	**0.023**	9 (8–9.25)	8 (7–9)	8 (8–8.5)	**0.007** ^f*^
Provide nutritional recommendations to support better nutritional status for patients, considering patient preferences	8 (8–9)	8 (8–9)	9 (8–10)	0.071	9 (8–10)	8 (7–9)	8 (8–8.5)	**0.007** ^f*^
Provide nutritional recommendations for discharged patients for formula drink (if clinically indicated and preferred) with instructions on how to prepare it	8 (8–9)	8 (7–9)	9 (8–10)	0.058	9 (8–10)	8 (7–9)	8 (6.5–9.25)	**<0.001** ^g*^
Evaluate and monitor a patient’s nutritional status at each follow-up visit	8 (7–9)	8 (7–9)	8 (8–9)	0.266	8 (8–10)	8 (7–9)	8 (7–8.5)	**<0.001** ^g*^
Provide basic nutritional advice (e.g., high energy, high protein diet education) for patients with cancer	9 (8–10)	9 (8–10)	9 (8–10)	0.894	9 (8–10)	8 (8–9)	8 (7.75–9.25)	**0.007** ^a*^
Provide nutritional recommendations for patients with a feeding tube	8 (8–9)	8 (7–9)	9 (8–10)	**0.017**	9 (8–10)	8 (7–9)	8 (7.75–9.25)	**<0.001** ^g*^
Troubleshoot individual nutrition impact symptoms and provide recommendations for my patients with cancer	8 (8–9)	8 (7–9)	9 (8–9.5)	**0.033**	9 (8–10)	8 (7–9)	8 (7.75–9.25)	**<0.001** ^g*^
Memory, attention and decision processes								
**Publication used for practice, *n* (%)**								
Hospital Nutrition Service Guidelines (Regulation of The Minister of Health of The Republic of Indonesia Number 78 Of 2013)	234 (87.3)	162 (89.5)	71 (82.6)	0.112	119 (85.0)	110 (90.9)	5 (83.3)	0.333
eNCPT (2020)	142 (53.0)	87 (48.1)	55 (64.0)	**0.015**	79 (56.4)	60 (49.6)	3 (50.0)	0.536
International Dietetics and Nutritional Terminology (IDNT) Reference Manual: Standard Language for the Nutrition Care Process 4th Edition (2013)	141 (52.6)	100 (55.2)	40 (46.5)	0.182	76 (54.3)	62 (51.2)	3 (50.0)	0.878
Technical guidelines on adult Palliative patients with cancer (Ministry of Health)	69 (25.7)	42 (23.2)	27 (31.4)	0.153	41 (29.3)	27 (22.3)	1 (16.7)	0.378
ESPEN guidelines on nutrition in cancer patients	100 (37.3)	57 (31.5)	42 (48.8)	**0.006**	65 (46.4)	35 (28.9)	0 (0)	**<0.001** ^h*, b**^
ESPEN practical guideline: Clinical Nutrition in cancer	84 (31.3)	52 (28.7)	31 (36.0)	0.227	55 (39.3)	29 (24.0)	0 (0)	**0.003** ^a*^
ASPEN Nutrition Guidelines for Adult Head and Neck Cancer: Protocol	57 (21.3)	30 (16.6)	26 (30.2)	**0.010**	36 (25.7)	21 (17.4)	0 (0)	0.061
Oncology Evidence-Based Nutrition Practice Guideline for Adults (Academy of Nutrition and Dietetics)	31 (11.6)	13 (7.2)	17 (19.8)	**0.002**	19 (13.6)	12 (9.9)	0 (0)	0.438
Position statement of cancer-related malnutrition and sarcopenia (Clinical Oncology Society of Australia)	16 (6.0)	3 (1.7)	12 (14.0)	**<0.001**	10 (7.1)	6 (5.0)	0 (0)	0.625
I am not aware of these publications	5 (1.9)	5 (2.8)	0 (0)	0.179	4 (2.9)	1 (0.8)	0 (0)	0.412
Other	14 (5.2)	11 (6.1)	3 (3.5)	0.559	7 (5.0)	6 (5.0)	1 (16.7)	0.447
Behavioral regulation								
**Preference for referral to a more experienced nutritionist or dietitian**								
If I receive a referral for an adult patient with cancer who is malnourished, I prefer to refer to a nutritionist or dietitian with more experience in this area	7 (5–9)	7.5 (5–9)	7 (5–8)	0.196	7 (5–9)	7 (5–8)	9 (6.5–10)	0.124
If I receive a referral for an adult patient with cancer who has comorbidities, e.g., diabetes, I prefer to refer to a nutritionist/dietitian with more experience in this area	7 (5–9)	8 (5–9)	7 (5–8)	0.189	8 (5–9)	7 (5–8)	9.5 (7.25–10)	**0.047**

eNCPT, electronic Nutrition Care Process Terminology; IDNT, International Dietetics and Nutritional Terminology; ESPEN, European Society for Parenteral and Enteral Nutrition; ASPEN, American Society for Parenteral and Enteral Nutrition; * significant difference is between participants working in metropolitan/large urban areas and regional/small cities; ** significant difference is between participants working in metropolitan/large urban areas and rural/remote; *** significant difference is between participants working in regional/small cities and rural/remote; ^a^ *p* = 0.008; ^b^ *p* = 0.034; ^c^ *p* = 0.014; ^d^ *p* = 0.003; ^e^ *p* = 0.012; ^f^ *p* = 0.005; ^g^ *p* < 0.001; ^h^ *p* = 0.004; Participants indicated level of agreement for each statement, 0 = ‘strongly disagree’, 10 = ‘strongly agree’; Numbers were described as median (interquartile range/IQR) from a 10-point Likert scale of agreement. Bold indicates *p* < 0.05.

**Table 3:** Practices and perceived barriers or enablers to the provision of nutrition care for patients with cancer by Indonesian nutritionists and dietitians: survey questions addressing domains of the TDF mapped to the opportunity aspect of the COM-B system.

Theoretical domain framework	All (*n* = 268)	Nutritionists/dietitians			Geographical location			
		Nutritionist (*n* = 181)	Dietitian (*n* = 86)	*p*-value	Metropolitan/large urban area (*n* = 140)	Regional or small city (*n* = 121)	Rural or remote (*n* = 6)	*p*-value
Social influences								
**Emotional support**								
I can rely on my nutrition/dietetic colleagues for emotional support in providing nutrition care to my patients with cancer	8 (8–9)	8 (8–9.25)	8 (8–9)	0.441	9 (8–9.5)	8 (8–9)	8 (7.75–10)	0.722
I can rely on my oncology team colleagues for emotional support in providing nutrition care to my patients with cancer	8 (8–9)	8 (7–9)	8 (8–9)	0.324	8 (8–9)	8 (7–9)	7.5 (7–10)	0.418
Environmental context and resources								
**Support available to manage a patient with cancer, *n* (%)**								
Peers	214 (79.9)	149 (82.3)	64 (74.4)	0.133	110 (78.6)	101 (83.5)	3 (50.0)	0.149
Mentor	40 (14.9)	31 (17.1)	9 (10.5)	0.154	22 (15.7)	18 (14.9)	0 (0)	0.572
Supervisor	58 (21.6)	43 (23.8)	14 (16.3)	0.164	37 (26.4)	21 (17.4)	0 (0)	**0.047**
Specialist oncology medical professionals	143 (53.4)	91 (50.3)	51 (59.3)	0.167	83 (59.3)	59 (48.8)	1 (16.7)	**0.039**
Medical professionals	156 (58.2)	106 (58.6)	49 (57.0)	0.806	73 (52.1)	78 (64.5)	4 (66.7)	0.119
Nursing team	191 (71.3)	124 (68.5)	66 (76.7)	0.165	95 (67.9)	95 (78.5)	1 (16.7)	**0.003** ^i**, d***^
Local hospital nutritionists and dietitians, doctors, or nurses (only select this if you do not work in a hospital)	96 (35.8)	75 (41.4)	21 (24.4)	**0.007**	56 (40.0)	35 (28.9)	5 (83.3)	**0.009** ^d***^
Online forums	50 (18.7)	26 (14.4)	24 (27.9)	**0.008**	33 (23.6)	16 (13.2)	1 (16.7)	0.097
Interest group email listings	11 (4.1)	6 (3.3)	5 (5.8)	0.337	7 (5.0)	4 (3.3)	0 (0)	0.613
Other	11 (4.1)	5 (2.8)	6 (7.0)	0.105	7 (5.0)	4 (3.3)	0 (0)	0.613
Barriers to providing nutrition care for patients with cancer, *n* (%)								
The time required to provide nutrition care for these patients exceeds my capacity	105 (39.2)	67 (37.0)	38 (44.2)	0.262	57 (40.7)	46 (38.0)	2 (33.3)	0.864
Lack of training in the area of patients with cancer	203 (75.7)	139 (76.8)	64 (74.4)	0.671	91 (65.0)	107 (88.4)	5 (83.3)	**<0.001** ^g*^
Training during working	85 (31.7)	56 (30.9)	29 (33.7)	0.648	47 (33.6)	36 (29.8)	2 (33.3)	0.801
Lack of multidisciplinary team support experienced in managing patients with cancer	94 (35.1)	63 (34.8)	31 (36.0)	0.843	47 (33.6)	45 (37.2)	2 (33.3)	0.826
Insufficient handover from referring medical professionals	55 (20.5)	34 (18.8)	20 (23.3)	0.395	32 (22.9)	21 (17.4)	1 (16.7)	0.529
Working with patients with cancer makes me anxious and stressed	15 (5.6)	10 (5.5)	5 (5.8)	1.000	8 (5.7)	5 (4.1)	1 (16.7)	0.379
Limited access to up-to-date research and guidelines on managing patients with cancer	115 (42.9)	75 (41.4)	40 (46.5)	0.434	61 (43.6)	51 (42.1)	3 (50.0)	0.917
Insufficient resources and tools for effective nutrition counseling	104 (38.8)	74 (40.9)	30 (34.9)	0.347	49 (35.0)	53 (43.8)	2 (33.3)	0.334
Lack of clear guidelines for the nutritional management of patients with cancer	58 (21.6)	43 (23.8)	15 (17.4)	0.242	25 (17.9)	31 (25.6)	2 (33.3)	0.252
Limited nutrition/dietetic staff	131 (48.9)	92 (50.8)	39 (45.3)	0.403	68 (48.6)	60 (49.6)	3 (50.0)	0.986
Frequent staff rotation	61 (22.8)	46(25.4)	15 (17.4)	0.147	35 (25.0)	26 (21.5)	0 (0)	0.165
Lack of equipment resources, e.g., computer access, electronic medical record	55 (20.5)	36 (19.9)	19 (22.1)	0.677	28 (20.0)	26 (21.5)	1 (16.7)	0.928
Difficult procedures for reimbursement of training fees	58 (21.6)	39 (21.5)	19 (22.1)	0.919	32 (22.9)	26 (21.5)	0 (0)	0.218
A part-time nutritionist/dietitian position	17 (6.3)	11 (6.1)	6 (7.0)	0.779	7 (5.0)	8 (6.6)	2 (33.3)	**0.021** ^j**^
Inappropriate salary according to qualification and experience	33 (12.3)	22 (12.2)	11 (12.8)	0.883	21 (15.0)	11 (9.1)	1 (16.7)	0.326
Distance to work from home is too far	8 (3.0)	6 (3.3)	2 (2.3)	1.000	7 (5.0)	1 (0.8)	0 (0)	0.095
Implementing a new licensing system (Law of The Republic of Indonesia Number 17, 2023 and Circular Letter Number HK.02.01/MENKES/6/2024)	26 (9.7)	21 (11.6)	5 (5.8)	0.136	17 (12.1)	9 (7.4)	0 (0)	0.317
Other	7 (2.6)	3 (1.7)	4 (4.7)	0.217	4 (2.9)	3 (2.5)	0 (0)	0.836
Enablers to providing nutrition care for patients with cancer, *n* (%)								
The time required to provide nutrition care for these patients is adequate	144 (53.7)	98 (54.1)	46 (53.5)	0.920	74 (52.9)	67 (55.4)	3 (50.0)	0.903
Easy/comfortable access to training	172 (64.2)	117 (64.6)	55 (64.0)	0.913	86 (61.4)	84 (69.4)	2 (33.3)	0.118
Day off for training – workload covered while training	95 (35.4)	61 (33.7)	34 (39.5)	0.352	47 (33.6)	46 (38.0)	2 (33.3)	0.751
Support from an experienced multidisciplinary team in managing patients with cancer is available	141 (52.6)	95 (52.5)	45 (52.3)	0.980	78 (55.7)	61 (50.4)	2 (33.3)	0.432
Dietitian referrals are available in a timely manner	49 (18.3)	30 (16.6)	19 (22.1)	0.276	31 (22.1)	17 (14.0)	1 (16.7)	0.236
Working with patients with cancer keeps me motivated	105 (39.2)	64 (35.4)	40 (46.5)	0.081	57 (40.7)	42 (34.7)	5 (83.3)	**0.047** ^k***^
Easy/comfortable access to up-to-date research and guidelines on managing patients with cancer	105 (39.2)	68 (37.6)	36 (41.9)	0.502	48 (34.3)	56 (46.3)	0 (0)	**0.007** ^l*, b***^
Adequate resources and tools for effective nutrition counseling	116 (43.3)	75 (41.4)	41 (47.7)	0.337	54 (38.6)	60 (49.6)	2 (33.3)	0.177
Clear hospital guidelines for nutrition care for patients with cancer	123 (45.9)	81 (44.8)	41 (47.7)	0.654	59 (42.1)	60 (49.6)	4 (66.7)	0.286
Adequate nutrition/dietetic staff.	122 (45.5)	81 (44.8)	41 (47.7)	0.654	63 (45.0)	57 (47.1)	2 (33.3)	0.777
No staff rotation (retain expertise)	46 (17.2)	33 (18.2)	13 (15.1)	0.529	32 (22.9)	14 (11.6)	0 (0)	**0.017** ^m*^
Adequate equipment resources, e.g., computer access, electronic medical records.	116 (43.3)	52 (28.7)	31 (36.0)	0.227	44 (31.4)	38 (31.4)	1 (16.7)	0.717
Simple procedures for reimbursement of training fee	43 (16.0)	28 (15.5)	14 (16.3)	0.865	18 (12.9)	22 (18.2)	3 (50.0)	**0.037** ^n**^
A full-time nutritionist/dietitian posit	50 (18.7)	33 (18.2)	17 (19.8)	0.764	31 (22.1)	18 (14.9)	1 (16.7)	0.318
Appropriate salary according to qualification and experience	64 (23.9)	43 (23.8)	20 (23.3)	0.928	32 (22.9)	28 (23.1)	3 (50.0)	0.365
Reasonable distance to work from home	32 (11.9)	21 (11.6)	10 (11.6)	0.995	15 (10.7)	15 (12.4)	2 (33.3)	0.243
Other	5 (1.9)	2 (1.1)	3 (3.5)	0.332	2 (1.4)	3 (2.5)	0 (0)	0.737
Resources for managing nutrition care								
I have adequate and accessible resources for managing nutrition care for patients with cancer (e.g., education materials, training, office space, access to electronic medical records, access to nutrition care forms)	8 (7–9)	8(7–9)	8(7–9.5)	0.683	8(8–10)	8(7–9)	8.5(8–9.25)	0.212

Bold indicates *p* < 0.05. *significant difference is between participants working in metropolitan/large urban areas and regional/small cities; ** significant difference is between participants working in metropolitan/large urban areas and rural/remote; *** significant difference is between participants working in regional/small cities and rural/remote; ^b^ *p* = 0.034;^d^ *p* = 0.003; ^g^ *p* = <0.001; ^i^ *p* = 0.018; ^j^ *p* = 0.045; ^k^ *p* = 0.026; ^l^ *p* = 0.048; ^m^ *p* = 0.017; ^n^ *p* = 0.039; Participants indicated level of agreement for each statement, 0 = ‘strongly disagree’, 10 = ‘strongly agree’; Numbers were described as median (interquartile range/IQR) from a 10-point Likert scale of agreement.

**Table 4:** Practices and perceived barriers or enablers to the provision of nutrition care for patients with cancer by Indonesian nutritionists and dietitians: survey questions addressing domains of the TDF mapped to the motivation aspect of the COM-B system.

Theoretical domain framework	All (*n* = 268)	Nutritionists/dietitians			Geographical Location			
		Nutritionist (*n* = 181)	Dietitian (*n* = 86)	*p*-value	Metropolitan/large urban area (*n* = 140)	Regional or small city (*n* = 121)	Rural or remote (*n* = 6)	*p*-value
Social/professional role and identity								
**Responsibility, role, and awareness**								
Nutritionists and dietitians play a crucial role in improving the daily lives of patients with cancer	10 (8.5–10)	10 (8–10)	10 (9–10)	0.827	10 (9–10)	9 (8–10)	9.5 (7.75–10)	**0.017** ^c*^
I am aware that other health professionals may be providing nutrition recommendations	9 (8–10)	9 (7–9.5)	9 (8–10)	0.222	9 (8–10)	8 (7–9)	9 (7–9.25)	**0.014** ^o*^
It is my responsibility to provide nutrition care to patients at risk of malnutrition	10 (9–10)	10 (9–10)	10 (9–10)	0.419	10 (9–10)	10 (9–10)	10 (8.75–10)	0.251
Beliefs about capabilities								
**Beliefs of the scope of practice for nutritionists and dietitians, *n* (%)**								
Decide the nutrition screening tool to be used	260 (97)	174 (96.1)	85 (98.8)	1.000	136 (97.1)	118 (97.5)	6 (100)	0.903
Conduct nutritional assessment through anthropometric measurements and interpretation of biochemical, clinical and dietary intake data for patients with cancer at risk of malnutrition	263 (98.1)	176 (97.2)	86 (100)	1.000	137 (97.9)	119 (98.3)	6 (100)	0.524
Identify nutrition problems, etiology, signs, and symptoms that formulate nutrition diagnosis for patients.	258 (96.3)	173 (95.6)	84 (97.7)	1.000	132 (94.3)	119 (98.3)	6 (100)	0.266
Determine nutritional intervention for patients with cancer	255 (95.1)	172 (95.0)	82 (95.3)	0.554	134 (95.7)	115 (95.0)	6 (100)	0.948
Monitor and evaluate the effect of nutritional intervention periodically	258 (96.3)	175 (96.7)	82 (95.3)	1.000	134 (95.7)	117 (96.7)	6 (100)	0.950
Provide nutritional recommendations for patients prior to discharge	254 (94.8)	175 (96.7)	78 (90.7)	0.524	133 (95.0)	114 (94.2)	6 (100)	0.205
Ensure that nutrition care is documented in the medical record	257 (95.9)	176 (97.2)	80 (93.0)	0.315	134 (95.7)	116 (95.9)	6 (100)	0.454
Provide nutrition care for outpatients with cancer	224 (83.6)	150 (82.9)	73 (84.9)	0.465	113 (80.7)	104 (86.0)	6 (100)	0.574
Determine the most appropriate tube feeding formula (if indicated) to meet a patient’s medical and nutrition needs.	254 (94.8)	172 (95.0)	81 (94.2)	1.000	132 (94.3)	115 (95.0)	6 (100)	0.470
Collaborate inter-professionally through oncology teams with nurses, medical professionals, and other health professionals to provide medical nutrition therapy	245 (91.4)	169 (93.4)	75 (87.2)	0.081	125 (89.3)	114 (94.2)	5 (83.3)	0.690
Confidence in ability								
Having medical and nursing support for my nutrition care practice would help me feel more confident.	9 (8.5–10)	9 (8–10)	10 (9–10)	0.267	10 (9–10)	9 (8–10)	9 (8.75–10)	0.066
Having more training in nutrition care practices in patients with cancer would help me feel more confident in this area	10 (9–10)	10 (9–10)	10 (9–10)	0.742	10 (9–10)	10 (9–10)	10 (8.75–10)	0.642
Hospital-based nutritionists and dietitians are best equipped to manage the nutrition care of patients with cancer	9 (8–10)	9 (8–10)	9 (8–10)	0.333	9 (8–10)	9 (8–10)	8.5 (8–9.25)	0.164
Nutritionists and dietitians are capable of providing nutrition care for patients with cancer	10 (9–10)	10 (9–10)	10 (9–10)	0.665	10 (9–10)	9 (9–10)	9.5 (8.5–10)	0.196
I know where to find best practice guidelines for the nutritional management of patients with cancer	8 (7–9)	8 (7–9)	8 (7–9)	0.103	8 (7–9)	8 (6–9)	8.5 (7.75–10)	**0.017** ^p*^
I have a clear plan under what circumstances I will recommend formula drinks to patients with cancer to increase overall intake.	9 (8–10)	8 (7.75–10)	9 (8–10)	0.314	9 (8–10)	8 (7–9)	8.5 (7.75–10)	**0.015** ^o*^
Beliefs about consequences								
**Potential repercussions of not providing nutrition care to patients with cancer who have complications and/or at risk of malnutrition, *n* (%)**								
Longer length of stay in the hospital	239 (89.2)	158 (87.3)	80 (93.0)	0.160	128 (91.4)	109 (90.1)	2 (33.3)	**<0.001** ^q**, r***^
More patients develop malnutrition	235 (87.7)	157 (86.7)	77 (89.5)	0.517	123 (87.9)	108 (89.3)	4 (66.7)	0.250
More patients have complex complications	217 (81.0)	148 (81.8)	68 (79.1)	0.600	111 (79.3)	100 (82.6)	5 (83.3)	0.779
Increased healthcare cost	195 (72.8)	130 (71.8)	64 (74.4)	0.657	105 (75.0)	88 (72.7)	2 (33.3)	0.111
Other	18 (6.7)	12 (6.6)	6 (7.0)	0.916	11 (7.9)	7 (5.8)	0 (0)	0.642
The importance of nutrition care								
Patients with cancer who do not receive adequate nutrition support are at risk of adverse consequences	10 (9–10)	10 (9–10)	10 (9–10)	0.064	10 (9–10)	10(9–10)	10 (7.75–10)	0.654
I see the value in providing nutrition care to patients with cancer	10 (9–10)	10 (9–10)	10 (9–10)	0.132	10 (9–10)	10(9–10)	9.5 (7–10)	**0.043**
Nutrition assessment and diagnosis are essential in providing person-centred nutrition intervention	10 (9–10)	10 (9–10)	10 (9–10)	0.662	10 (9–10)	10(9–10)	9 (8–10)	0.087
Intentions								
**Intention to learn and manage more patients**								
I would like to manage more patients with cancer in a clinical setting	9 (8–10)	9 (8–10)	9 (8–10)	**0.016**	9 (8–10)	9(8–10)	8 (7.75–9.25)	0.163
I would like to learn more about nutrition care for patients with cancer, including patients with complex comorbidities and malnutrition	10 (9–10)	10 (9–10)	10 (9–10)	0.582	10 (9–10)	10(9–10)	9 (8–10)	0.280
Goals								
**Learning/development opportunities planned to be completed within the next 12 months, *n* (%)**								
Online workshop on advanced nutrition care for patients with cancer	147 (54.9)	96 (53.0)	51 (59.3)	0.336	85 (60.7)	59 (48.8)	3 (50.0)	0.148
Webinar featuring case studies and real-world scenarios in managing patients with cancer	205 (76.5)	137 (75.7)	68 (79.1)	0.541	100 (71.4)	101 (83.5)	4 (66.7)	0.057
Certification program for specialized skills in managing patients with cancer	136 (50.7)	86 (47.5)	50 (58.1)	0.105	84 (60.0)	51 (42.1)	1 (16.7)	**0.003** ^m*^
Interactive virtual simulations for hands-on experience in malnourished patients with cancer, i.e., patients with tube feeding	88 (32.8)	58 (32.0)	30 (34.9)	0.645	49 (35.0)	39 (32.2)	0 (0)	0.079
Access to a comprehensive online resource library with the latest research and nutritional guidelines for managing patients with cancer	98 (36.6)	63 (34.8)	35 (40.7)	0.351	55 (39.3)	43 (35.5)	0 (0)	0.051
Regional meetings to network and share experiences with other professionals in the related field	88 (32.8)	57 (31.5)	31 (36.0)	0.459	48 (34.3)	38 (31.4)	2 (33.3)	0.885
Mentorship program connecting nutritionists and dietitians for ongoing guidance in managing patients with cancer	128 (47.8)	87 (48.1)	41 (47.7)	0.952	64 (45.7)	60 (49.6)	3 (50.0)	0.817
Collaborative research opportunities with supporting funding in the field of nutrition care for patients with cancer	91 (34.0)	56 (30.9)	35 (40.7)	0.116	59 (42.1)	32 (26.4)	0 (0)	**0.002** ^a*^
Other	5 (1.9)	4 (2.2)	1 (1.2)	1.000	2 (1.4)	3 (2.5)	0 (0)	0.737
Plan to access training								
I have a clear plan to access training so that I can be more effective in delivering nutrition care	8 (7–10)	8(7–10)	8(8–9)	0.614	9(8–10)	8 (7–9)	8 (7–9.25)	**0.012** ^s*^
Reinforcement								
**Satisfaction and recognition**								
I feel a sense of personal satisfaction whenever patients indicate that they are pleased with the nutrition care that I provide	10 (9–10)	10 (9–10)	10 (9–10)	0.717	10 (9–10)	10 (9–10)	9.5 (7–10)	**0.049**
I receive recognition from my colleagues whenever patients indicate that they are pleased with the nutrition care that I provide	9 (8–10)	9 (8–10)	9 (8–10)	0.549	9 (8–10)	9 (8–10)	8.5 (8–9.25)	0.338
Emotion								
**Feeling overwhelmed, enjoyment and respected**								
As a nutritionist/dietitian working with patients with cancer, have you felt overwhelmed while doing your day-to-day work during the past two weeks?	5 (2–8)	5 (2–8)	5 (2–8)	0.625	5 (2–8)	5 (2–7)	5 (0–7.5)	0.538
As a nutritionist/dietitian working with patients with cancer, have you been able to enjoy your normal day-to-day activities during the past two weeks?	8 (7–10)	8 (7–10)	8 (7–10)	0.446	8 (7–10)	8 (7–10)	8 (6.25–10)	0.687
I am a respected member of the oncology team.	7 (5–8)	7(5–8)	8 (5–8)	**0.016**	7 (5–8)	7 (5–8)	7 (6.5–8.5)	0.618

Bold indicates *p* < 0.05. *significant difference is between participants working in metropolitan/large urban areas and regional/small cities; ** significant difference is between participants working in metropolitan/large urban areas and rural/remote; *** significant difference is between participants working in regional/small cities and rural/remote; ^a^ *p* = 0.008; ^c^ *p* = 0.014; ^m^ *p* = 0.004; ^o^ *p* = 0.011; ^p^ *p* = 0.030; ^q^ *p* = 0.001; ^r^ *p* = 0.002; ^s^ *p* = 0.010; Participants indicated level of agreement for each statement, 0 = ‘strongly disagree’, 10 = ‘strongly agree’; Numbers were described as median (interquartile range/IQR) from 10-point Likert scale of agreement.

#### 3.2.1 Capability: physical and psychological

Overall, participants demonstrated limited awareness of nutrition care guidelines (TDF domain: Knowledge), except for national hospital nutrition guidelines published by the Indonesian Ministry of Health (Table 2). Awareness of oncology-specific nutrition guidelines ranged from 11.2% (Clinical Oncology Society of Australia (COSA)) to 44.8% (European Society for Parenteral and Enteral Nutrition (ESPEN)), resulting in limited application in clinical practice (TDF domains: Memory, attention, decision processes). Dietitians reported significantly greater awareness of nutrition care guidelines compared to nutritionists: ESPEN ((53.5% vs. 39.2%) (32) and 52.3% vs. 40.9%) (33), the American Society for Parenteral and Enteral Nutrition (ASPEN) (38.4% vs. 22.1%) (34) the Academy of Nutrition and Dietetics (25.6% vs. 10.5%) (35), and COSA (22.1% vs. 5.5%) (36).

Confidence in providing nutrition care was high, particularly in giving basic nutritional advice (e.g., high energy, high protein diet education) for patients with cancer (median 9) (8–10)). Those working in metropolitan areas reported significantly higher confidence than those working in small cities in conducting nutrition assessment (*p* = 0.003), providing recommendations for patients with feeding tubes (*p* < 0.001), and troubleshooting management of nutrition impact symptoms (*p* < 0.001). Dietitians were significantly more confident than nutritionists in each of these components of practice (*p* = 0.006, *p* = 0.017, *p* = 0.033, respectively); (however, absolute median differences were modest (e.g., median 9 vs. 8). Most participants routinely completed individual components of nutritional assessment (e.g., anthropometric (91.8%), biochemical (88.4%), physical (71.3%) and dietary intake data (95.1%)); however, only 55.6% indicated using this information to complete a formal malnutrition assessment using a validated tool. Only 50% considered the impact of cancer treatment and medications (TDF domain: Skill) when completing a nutrition assessment.

#### 3.2.2 Opportunity: physical and social

Participants reported being supported predominantly by peers (79.9%), the nursing team (71.3%), medical professionals (58.2%), specialist oncology medical professionals (53.4%), and local hospital clinicians (35.8%) (Table 3). Across geographical locations and the professions, participants indicated adequate access to general resources, including educational materials, general nutrition training, office space, and electronic medical records and forms. The most frequently reported barriers to providing nutrition care according to the NCP were a lack of oncology-specific nutrition care training (75.7%) (particularly for those in regional or remote areas), limited nutrition/dietetic staff (48.9%), and limited access to up-to-date research and clinical guidelines for managing patients with cancer (42.9%). Enablers to providing nutrition care for patients with cancer included accessible training (64.2%), adequate time to deliver nutrition care (53.7%), and support from an experienced multidisciplinary team (52.6%) (TDF domain: Environmental context and resources). Overall, participants reported feeling emotionally supported by nutrition/dietetic and oncology team colleagues in providing nutrition care for patients with cancer (TDF domain: Social influences).

#### 3.2.3 Motivation: reflective and automatic

Participants reported a strong professional identity with high confidence in their abilities (median 10 (8.5–10)) and a high sense of responsibility for providing nutritional care of patients at risk of malnutrition (median 10 (9, 10)), regardless of profession (Table 4). They had high awareness of the importance of the NCP and understood that a failure to implement the NCP could lead to adverse consequences such as longer length of hospital stay (89.2%), development of malnutrition (87.7%), complications (81%), and increased healthcare costs (72.8%) (TDF domain: Beliefs about consequences) (Table 4). Those working in metropolitan areas had a significantly higher level of confidence in their professional role compared to those in regional or small cities (*p* = 0.017) (TDF domain: Social/professional roles and identity). Similar findings were observed regarding participants’ ability to locate best practice guidelines (*p* = 0.017) and recommend formula drinks (*p* = 0.015) (TDF domain: Beliefs about capabilities), although group median differences remained small despite statistical significance. Most participants believed that their scope of practice encompassed all elements of nutrition care, with similar findings reported by both professional and geographical location subgroups. Feelings of satisfaction and recognition were reported when patients expressed appreciation for the nutrition care provided (TDF domain: Reinforcement). Dietitians identified more strongly as respected members of the oncology team, compared with nutritionists (*p* = 0.016).

**Figure 3:**
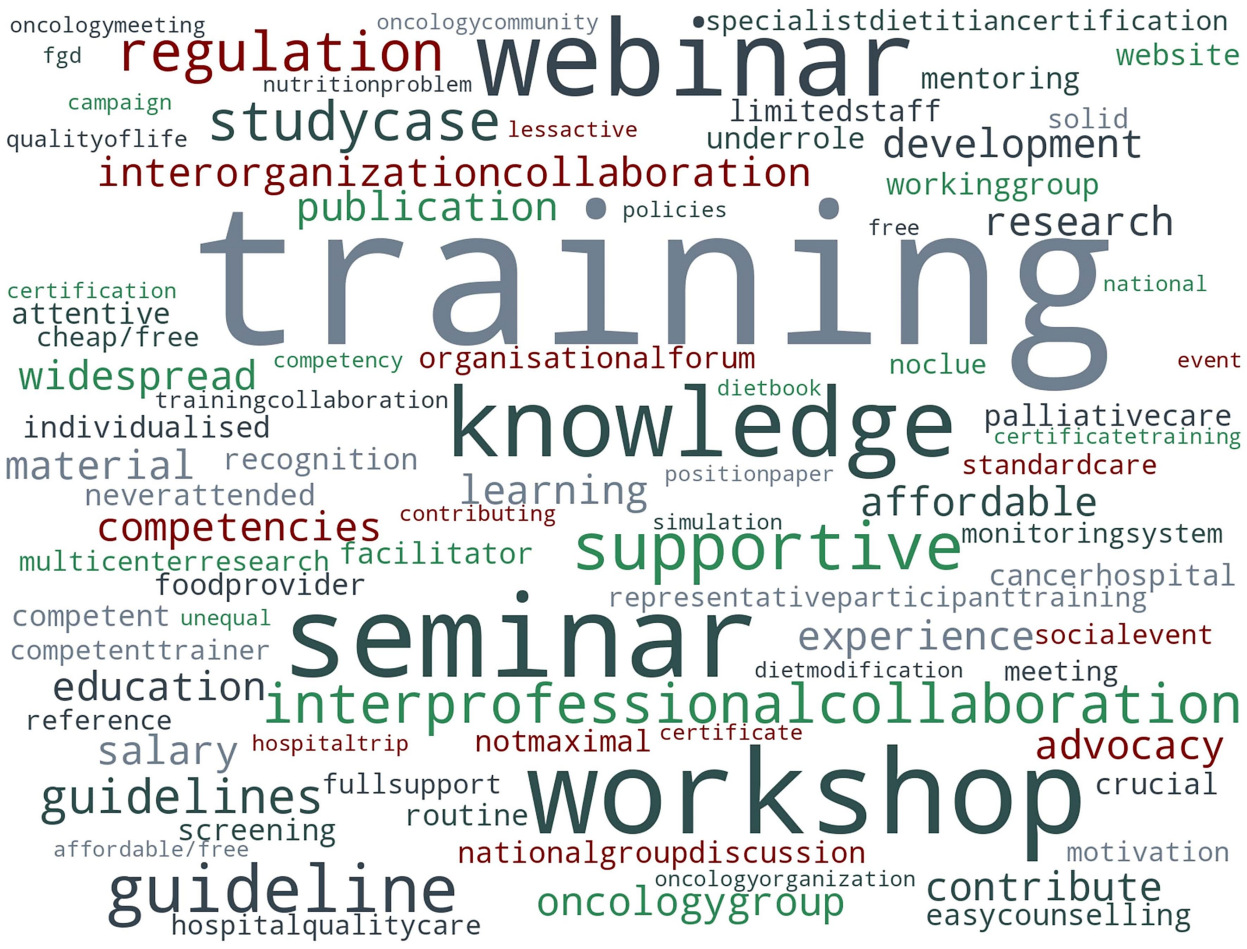
Visual overview of participants’ comments regarding potential helpful contributions by the Indonesian Dietitians Association.

Participants identified several preferred formats for future learning and professional development including case studies (76.5%), online workshops (54.9%), and certification programs (50.7%) (TDF domain: Goals). Most participants (87.7%) responded to an open-ended question regarding how the Indonesian Dietitian Association could enhance nutrition care for patients with cancer (Figure 3). The term ‘training’ featured prominently in responses, alongside familiar formats such as a ‘workshop’, ‘seminar’, or ‘webinar’.

## 4 Discussion

This is the first study to explore current oncology nutrition care practices in Indonesia, and identify barriers and enablers using behavioral frameworks (TDF and COM-B). Findings reveal limited awareness of international oncology nutrition care guidelines, and low uptake of validated malnutrition assessment tools despite participants reporting high confidence and strong professional identity. Dietitians demonstrated greater confidence than nutritionists in conducting nutrition assessments, providing tube-feeding recommendations, and managing nutrition impact symptoms. The most frequently reported barrier to providing nutrition care according to the NCP was the lack of oncology-specific training, despite general nutrition training being widely accessible.

A limited number of studies have examined general nutrition care practices in Indonesia. A study of dietitians’ perceptions of the NCP showed that 62% of nutritionists and dietitians held good or positive perceptions of nutrition care which was associated with their level of nutrition education (37). The number of components in the NCP and the volume of standardized NCP terminologies were identified as barriers to the implementation of nutrition care (37). In another Indonesian study, barriers identified were a lack of both communication during interprofessional collaboration and access to training for some dietitians (38). International evidence also demonstrates variability in NCP implementation. A multinational survey of dietitians (n = 6,719) across 10 countries found substantial differences in the use of the NCP, with Australia, New Zealand, and the United States reporting higher implementation rates than several European countries (Canada, Denmark, Greece, Ireland, Norway, Sweden, and Switzerland). Successful implementation was associated with practitioner experience and the presence of clear strategies (39). A 2020 follow-up study identified lack of time as the most common barrier and endorsement by national dietetic associations as the most common enabler (16). Although these studies investigated implementation, attitudes, and knowledge regarding the NCP in general patient care (16, 37–40), they did not employ a theoretical framework to help understand the underlying behavioral factors that influence practice.

Nearly half of the participants reported limited access to research and oncology-specific nutrition guidelines due to language barriers and the lack of Indonesian-language resources. International evidence-based oncology nutrition guidelines, including those published in Europe (32, 33), the United States of America (34, 35) and Australia (36) are essential for informing multidisciplinary teams (including nutritionists and dietitians) in the clinical management of patients with cancer, and also contribute to optimal resource management and service delivery (36). However, most are only available in English, creating a significant barrier for non-English speaking practitioners which may lead to miscommunication, frustration, and hinder decision-making (41). These findings align with our recent scoping review of oncology nutrition care practices in Southeast Asia (14), which identified limited country-specific clinical guidelines or protocols as a key challenge. A 2026 scoping review of international practice similarly reported that 17 of 19 studies cited a lack of resources as the most common barriers to timely nutrition support in patients with cancer, with several calling for more detailed guidelines and noting that generic cancer guidelines do not account for cancer-specific needs, often leaving individual centres responsible for developing their own protocols (42). They are also in agreement with a multinational survey of dietitians across five European centres where a key barrier to nutrition care in head, neck and oesophageal cancer included lack of standardized protocols (40).

Survey questions regarding awareness of oncology nutrition guidelines and the choice of publications used to guide practice relate to the TDF domains of ‘Knowledge’ and ‘Memory, attention and decision process’, both of which fall within the ‘psychological capability’ component of the COM-B system. Clinicians often rely on experience and contextual factors, including guideline familiarity to inform clinical practice, highlighting the role of memory and decision-making processes (43). Currently, Indonesia has only one guideline specific to palliative cancer care published by the Ministry of Health (44), used by 25.7% of participants. To strengthen the capability of Indonesian nutritionists and dietitians to deliver evidence-based nutrition care, there is a need for comprehensive evidence-based nutrition guidelines in the Indonesian language that are culturally adapted, concise, free and easy to access (17). These findings have clear policy implications and provide a strong rationale for the Ministry of Health, in partnership with the national cancer board and the Indonesian Dietetics Association, to establish an expert working group to develop national oncology nutrition care guidelines.

The survey results suggest that Indonesian nutritionists and dietitians have the skills to provide nutrition care according to the NCP, including completing elements of nutritional assessments such as dietary intake, anthropometry and biochemical data review. However, only 57% of the participants used a validated malnutrition assessment tool and considered the effects of treatment and medication. Malnutrition assessment tools are essential for diagnosing malnutrition, guiding re-assessment and evaluating the outcomes of nutrition intervention (32, 33, 35, 36). Tools such as Subjective Global Assessment (45), Patient-Generated Subjective Global Assessment (46), and Global Leadership Initiative on Malnutrition (47) are widely used in international clinical practice but require training for accurate application (48–50). This practice gap in the first step of the NCP suggests a need for targeted training and skill development (physical and psychological capability) to ensure comprehensive assessment and monitoring through a patient’s cancer journey (18, 51). Future work is needed to facilitate the implementation of those tools into clinical practice by nutrition professionals in Indonesia.

Training emerged as a key enabler for increasing confidence in oncology nutrition care. Continuous professional development is essential to equip practitioners with the knowledge and skills required for evidence-based oncology nutrition care (37). The Indonesian Dietitian Association plays a pivotal role in organizing training programs and providing qualified facilitators. Currently, a variety of accessible training formats in general nutrition care exists, including online and in-person sessions, offering flexibility in content, facilitators and cost. However, 75.7% of participants reported that the major barrier was limited availability of oncology-specific nutrition training, aligning with the ‘Environmental context and resources’ TDF domain, and the ‘Opportunity’ component of the COM-B system. It highlights the need for targeted professional development initiatives to update practitioners’ knowledge and skills for managing patients with cancer. Addressing this barrier requires diverse, accessible formats such as webinars, online workshops and blended learning approaches, which have been shown to be cost-effective, feasible, acceptable and effective in improving knowledge and skills among health professionals in low and middle-income countries (52–54). An Iranian (2020) and an American study (2022) similarly reported that participants preferred webinars as their primary mode of training, followed by online workshops (53, 54). Expanding access to oncology-specific training through these modalities is a practical strategy to overcome the current gaps and support the professional growth of nutritionists and dietitians. A further policy recommendation is for the Association of Nutrition Higher Education of Indonesia to strengthen the oncology nutrition content within dietetics education curricula, ensuring that future graduates enter the workforce with foundational competencies in cancer care. In parallel, the Indonesian Dietetics Association could support regular and accessible oncology nutrition care professional development.

A further barrier identified was the impact of Indonesia’s 2023 licensing system (55), which mandates that all nutritionists and dietitians hold a dietetics degree to maintain registration. This reform marks a significant shift in the evolution of the nutrition and dietetics professions in Indonesia. Prior to 2023, practitioners were categorized as technical registered nutritionists, dietitians, and registered dietitians based on their level of education (13, 56). The new requirement affects nutritionists with qualifications limited to an undergraduate nutrition degree. Further 2024 government regulations (57–59) allow nutritionists to continue practising until their existing licenses expire (within five years), during which time they must complete a dietetics degree. Although the majority use Recognition of Prior Learning to convert work experience into academic credit, balancing employment with additional study may contribute to mental stress, and a risk of burnout (60, 61). To facilitate this transition, some hospitals have introduced local measures to allow nutritionists to work under the supervision of licensed dietitians. In this study, nutritionists were less confident than dietitians in conducting nutritional assessment, identifying malnutrition-related signs and symptoms, providing recommendations to address nutritional problems including the use of a feeding tube, and troubleshooting individual nutrition impact symptoms. Ultimately, this licensing reform aims to improve the quality of nutrition care provided by practitioners and outcomes for patients in Indonesia.

Although artificial intelligence was not a focus of this study, emerging digital and AI-enabled decision support tools may offer future opportunities to address some of the identified barriers in resource-constrained settings, particularly with limited staffing. While nutritionists and dietitians remain essential for delivering personalized oncology nutrition care, integrating AI can streamline routine tasks such as assessments and meal monitoring, allowing clinicians to devote more time to advanced decision-making and in-depth patient counseling (62).

## 5 Strength and limitation

A key strength of this novel study is the application of behavioral theoretical frameworks (TDF and COM-B) to systematically explore oncology nutrition care practices among Indonesian nutritionists and dietitians. Mapping survey questions to these frameworks provides actionable insights for designing future implementation strategies that address the underlying barriers and enablers influencing Indonesian nutrition care practice.

However, several limitations should be acknowledged. First, the generalisability of findings to other clinical populations or countries may be limited due to differences in healthcare systems, professional training and scope of practice among nutritionists and dietitians. Participants were recruited through convenience sampling, which may result in a sample with shared characteristics and may not fully capture the diversity of the broader workforce. Although 91% of the participants were female, this reflects the gender distribution of the Indonesian nutritionist and dietitian workforce. Most respondents resided in Java and Bali; however, the relatively large sample size (n = 268) and representation from metropolitan, regional, and rural areas enhance the relevance of the findings within the Indonesian context. The use of an online survey limited opportunities to clarify questions in real time, although providing the primary researcher’s WhatsApp contact enabled respondents to seek clarification when needed. To minimize potential social desirability bias where participants may report ideal rather than actual practices, the survey included a clear introductory explanation, ensured anonymity, and used neutral, non-judgmental wording that had been reviewed by Indonesian and Australian nutritionists and dietitians. As the data was self-reported, there is also risk of response bias and possible overestimation of confidence or nutrition care practice.

## 6 Conclusion

This study highlights critical gaps in oncology nutrition care in Indonesia. While nutritionists and dietitians report high confidence in delivering nutrition care and routinely perform individual components of the NCP, comprehensive nutrition assessment using a validated tool is not standard practice. Limited access to international oncology nutrition guidelines, most of which are only available in English, and the lack of oncology-specific nutrition care training further hinder evidence-based nutrition care. To address these gaps, there is a need for culturally adapted Indonesian language evidence-based nutrition care guidelines, implementation of validated nutrition assessment tools into routine practice, and accessible cost-effective oncology-specific nutrition training. These strategies are essential to strengthen capability, opportunity and motivation for evidence-based practice and ultimately improve patient outcomes. Future research should evaluate the effectiveness of these interventions and explore scalable models for other upper-middle or low-middle-income countries facing similar challenges.

## Data Availability

The original contributions presented in the study are included in the article/supplementary material, further inquiries can be directed to the corresponding author.
